# Optimal fluid management strategies in patients with heart failure: a systematic review and meta-analysis of randomized controlled trials

**DOI:** 10.3389/fcvm.2025.1636862

**Published:** 2025-10-31

**Authors:** Umar G. Adamu, Blessing Muponda, Nqoba Tsabedze

**Affiliations:** Division of Cardiology, Department of Internal Medicine, School of Clinical Medicine, Faculty of Health Sciences, University of the Witwatersrand, Johannesburg, South Africa

**Keywords:** heart failure, fluid restriction, liberal fluid intake, hospitalization, mortality, quality of life

## Abstract

**Background:**

Fluid restriction is frequently recommended in heart failure (HF) management to prevent volume overload and improve clinical outcomes. However, the evidence supporting this practice remains limited. This meta-analysis aimed to evaluate the impact of fluid restriction vs. liberal fluid intake on clinical and patient-centered outcomes in individuals with HF.

**Methods:**

A systematic search of PubMed, Embase, Cochrane Library, and ClinicalTrials.gov was conducted through April 27, 2025, to identify randomized controlled trials (RCTs) comparing restrictive and liberal fluid strategies in HF. Pooled risk ratios (RRs) for binary outcomes and weighted mean differences (WMDs) for continuous outcomes with 95% confidence intervals (CIs) were calculated using a random-effects model.

**Results:**

Four RCTs with a total of 747 patients were included, of whom 378 (50.6%) were randomized to liberal fluid intake. There were no significant differences between groups regarding all-cause mortality (RR: 1.71; 95% CI: 0.37–3.72; *p* = 0.27), HF rehospitalization (RR: 0.71; 95% CI: 0.46–1.10; *p* = 0.13) or thirst (WMD: 4.78; 95% CI: −6.72 to −16.28; *p* = 0.42). Patients in the fluid restriction group had significantly lower fluid intake (WMD: −361.84 mL/day; 95% CI: −552.89 to −170.78; *p* < 0.001) and lower adherence (WMD: 16.47; 95% CI: 6.45–26.50; *p* = 0.001). No significant differences were observed between groups in terms of acute kidney injury, weight loss, or patient-reported quality of life, and Kansas City Cardiomyopathy Questionnaire (KCCQ) Clinical summary score.

**Conclusions:**

In this meta-analysis, fluid restriction significantly reduced total fluid intake but did not improve clinical outcomes in patients with HF. Adherence was higher with liberal fluid intake. These findings support an individualized approach to fluid management in patients with HF.

**Systematic Review Registration:**

PROSPERO CRD420251048914.

## 1 Introduction

Heart failure (HF) is a progressive clinical syndrome characterized by the heart's inability to pump sufficient blood to meet the body's metabolic demands, resulting in symptoms including dyspnoea and fluid retention (1). As the global incidence and prevalence of HF continue to rise, it remains a leading cause of hospitalization and mortality, posing a substantial public health burden (2). One of the central challenges in HF management is achieving an optimal fluid balance (3, 4). Therefore, determining whether a liberal or restrictive fluid strategy yields better outcomes continues to challenge both clinicians and researchers.

Fluid restriction is commonly recommended, particularly in patients with advanced HF or hyponatremia, to reduce the risk of volume overload, clinical deterioration, and rehospitalization. Some studies suggest that a liberal fluid intake may enhance patient comfort and hydration status without adversely affecting clinical outcomes, while others highlight the potentials risks of renal dysfunction and volume overload associated with more liberal intake (5–7). Current guidelines from the European Society of Cardiology and American Heart Association/American College of Cardiology recommend fluid restriction only for selected symptomatic patients, without strong supporting evidence (2, 8).

Previous meta-analyses have found no significant difference in mortality or rehospitalization between liberal and restrictive strategies, although their conclusions were limited by small sample sizes and methodological heterogeneity (9, 10). Moreover, many of these studies incorporated concurrent sodium restriction, complicating the interpretation of fluid management effects alone. Recently, the FRESH-UP RCT compared liberal and restrictive fluid intake in compensated chronic HF patients, providing new data that may improve the power of pooled analyses (11). In light of this, we performed an updated systematic review and meta-analysis to assess the efficacy and safety of liberal vs. restrictive fluid intake strategies in patients with HF.

## 2 Materials and methods

### 2.1 Study design and search strategy

This systematic review and meta-analysis was designed and conducted in accordance with the Preferred Reporting Items of Systematic Reviews and Meta-Analysis (PRISMA) guideline (12). This study was registered in the International Prospective Register of Systematic Reviews (PROSPERO) under the protocol number CRD420251048914. Two authors (U.G.A. and B.M.) systematically searched PubMed, Embase, Cochrane Central Register of Controlled Trials, and ClinicalTrials.gov for eligible studies from inception to April 2025. The search terms included heart failure, fluid intake, fluid therapy, fluid management, water intake, increase fluid intake, liberal fluid, restricted fluid, fluid restriction and RCT. The complete search strategies are provided in Supplementary Table 1. The references from all the included studies and reviews were also searched manually.

### 2.2 Inclusion and exclusion criteria

Studies were eligible for inclusion if they met the following criteria: (1) randomized controlled trials (RCTs); (2) compared restrictive vs. liberal fluid intake; (3) included patients with chronic HF, with or without acute decompensation; and (4) reported at least one of the pre-defined outcomes of interest. Studies were excluded if they (1) lacked a control group; (2) had no outcome of interest; (3) included sodium restriction; and (4) were editorials, conference abstracts, case reports, or observational studies.

### 2.3 Data extraction

Two authors (U.G.A. and B.M.) independently extracted data using pre-defined criteria. Extracted baseline characteristics included year of publication, country, study design, age, sex, sample size, type of HF, ejection fraction (EF), and duration of follow-up. Any discrepancies were resolved by consensus, with adjudication by the senior author (N.T.) when required.

### 2.4 Outcomes and subgroup analyses

The outcomes of interest included: (1) all-cause mortality, (2) HF rehospitalization, (3) thirst, (4) total fluid intake/day, (5) weight change, (6) Kansas City Cardiomyopathy Questionnaire overall summary score (KCCQ-OSS), (7) KCCQ Clinical summary score (KCCQ-CSS), (8) quality of life (QoL), (9) mean serum sodium, (10) mean serum creatinine, (11) adherence, and (12) change in the dose of loop diuretics.

Subgroup analyses were conducted based on (1) EF; (2) HF status (compensated vs. decompensated); and (3) the degree of daily fluid restriction.

### 2.5 Quality assessment

Two authors (U.G.A. and B.M.) independently assessed risk of bias in the included randomized trials using the Cochrane Risk of Bias tool (RoB 2) (13). Any disagreements were resolved by consensus or consultation with the third reviewer. Publication bias was assessed with funnel-plot analysis of daily fluid intake and thirst endpoint to evaluate the symmetric distribution of trials with similar weights. We also performed leave-one-out sensitivity analysis for all outcomes to ensure stability of the pooled treatment effect.

### 2.6 Statistical analysis

We used the DerSimonian and Laird random-effects model for all outcomes. Risk ratios (RRs) for binary endpoints and weighted mean differences (WMDs) for continuous endpoints with 95% confidence intervals (CIs) were computed. Heterogeneity was examined with Cochran's *Q* test, Higgin's *I*^2^ statistics, and T^2^ statistics; *p* values <0.10 and *I*^2^ > 50% were considered significant for heterogeneity. The guidelines of the Cochrane Handbook for Systematic Reviews of Interventions were used for data handling (14). *p*-values of < 0.05 were considered statistically significant. All statistical analyses were performed using RevMan 5.4.1 (Nordic Cochrane Centre, The Cochrane Collaboration, Copenhagen, Denmark).

## 3 Results

### 3.1 Study selection and characteristics

**Figure 1:**
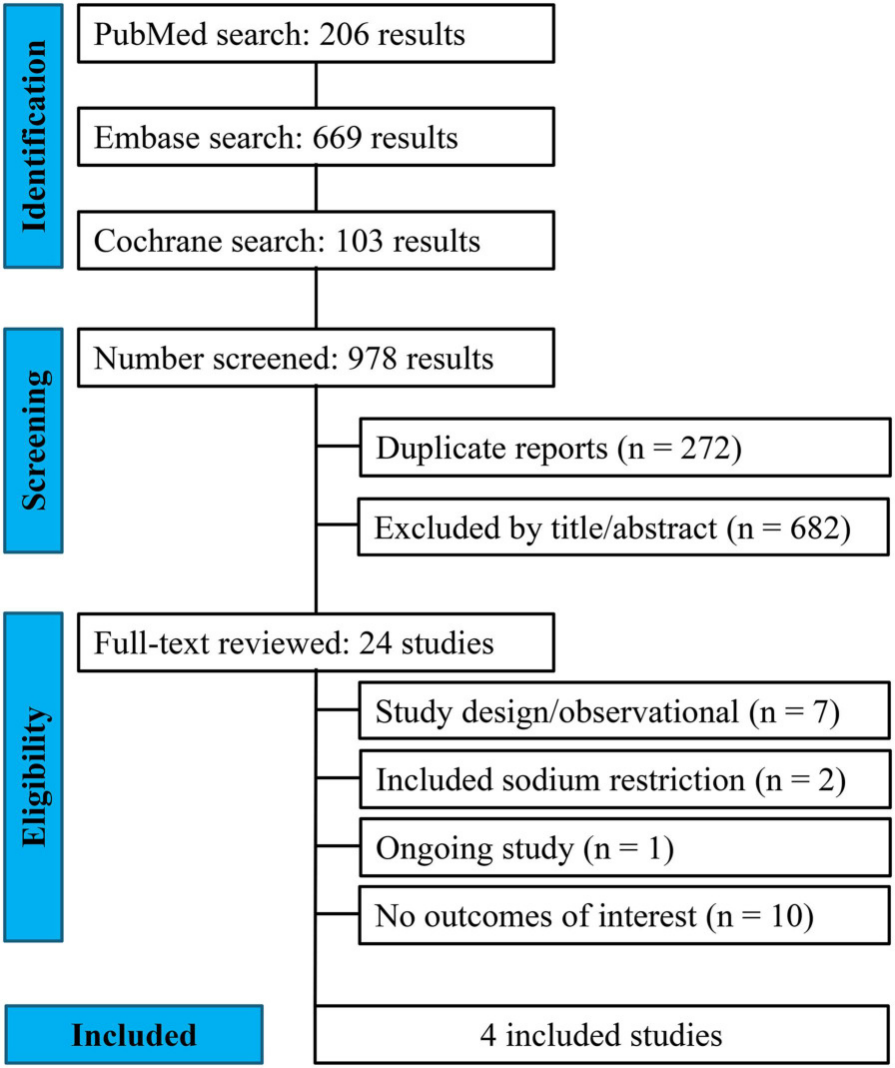
PRISMA flow diagram of study screening and selection.

Our systematic search yielded 978 potential articles (Figure 1). After removing duplicate records and studies based on title/abstract, 24 studies were assessed for eligibility. Of these, four RCTs met the inclusion criteria (5–7, 11). A total of 747 patients were included, of whom 378 (50.6%) were allocated to the liberal fluid intake group. The mean age ranged from 62.5 to 75 years, and the follow-up duration varied from 2 to 112 days. The weighted mean age of participants across the included trials was 68.9 ± 11.5 years (range 62.5–75 years), with follow-up durations varying from 2 to 112 days. The weighted mean left ventricular EF was 33.9 ± 12.2% (range 21.6–40.3%), and the proportion of male participants ranged from 38.7% to 67.6%. Table 1 summarizes the main characteristics of the included studies.

**Table 1:** Baseline characteristics of included studies.

Study variables	Travers 2007 LFI/FRI	Holst 2008 LFI/FRI	SALT-HF 2013 LFI/FRI	FRESH-UP 2025 LFI/FRI
Sites, Country	1, Ireland	2, Sweden	1, USA	7, The Netherlands
No. of participants	33/34	65/65	26/20	254/250
Age, years^a^	73/75	70/70	63.2/61.4	69.4/69.0
Male, n (%)	16 (49)/20 (59)	54 (83)/54 (83)	16 (61.3)/8 (38.7)	170 (66.9)/169 (67.6)
BMI, Kg/m^2^^a^	NA	NA	29.8/27.8	28.4/27.9
Ischaemic HF, *n* (%)	19 (59)/25 (76)	48 (74)/48 (74)	3(11.5)/5(25)	108 (42.5)/113 (45.2)
Fluid intake, mL/day^b^	1466.6/1074.3	1955/1479	NA	1764/1480
NYHA (III), *n* (%)	NA/NA	5 (9)/6 (8)	13 (50)/15 (75)	36 (14.2)/29 (11.6)
Mean LVEF^a^, %	40.2/37.4	NA	21.6/24.0	40.3/40.2
Frusemide, mg/day	74/76^a^	NA	98/138^b^	40/40^b^
Follow-up, days	2	112	60	90

a Mean and standard deviation.

b Median with interquartile range; ACEI/ARBs: angiotensin converting enzyme inhibitors/angiotensin receptor blockers; BMI, body mass index; FRI: fluid restricted; LFI; liberal fluid intake; LVEF: left ventricular ejection fraction; NA: not available; NYHA: New York heart association.

### 3.2 Outcomes

**Figure 2:**
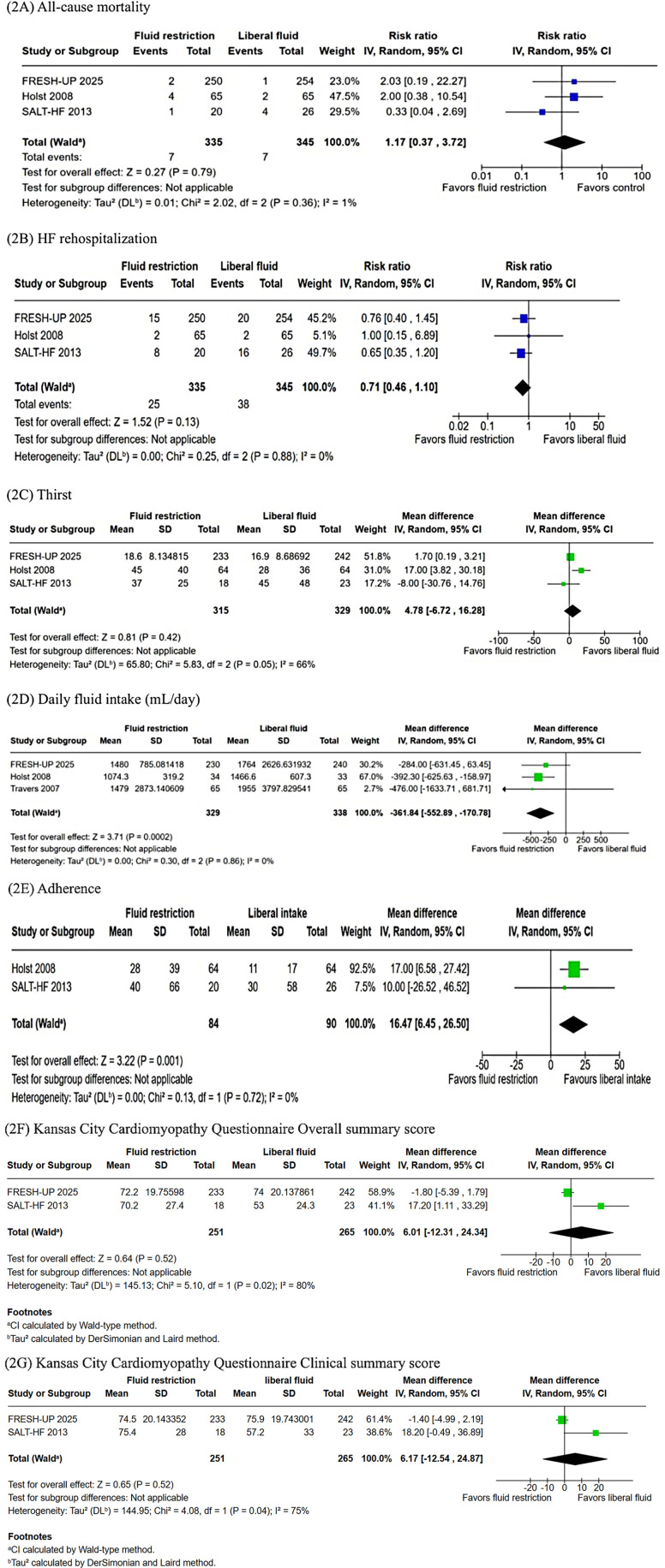
**(A)** The incidence of all-cause mortality was not significantly different between groups (*p* = 0.39). **(B)** The incidence of HF rehospitalization was same between groups (*p* = 0.13). **(C)** There was no difference between groups in the incidence of thirst (*p* = 0.42). **(D)** The total fluid intake (mL/day) was significantly lower in the FRI group (*p* < 0.001). **(E)** The incidence of adherence was significantly higher in the LFI group (*p* = 0.001). **(F)** The Kansas City cardiomyopathy questionnaire overall summary score was not different between groups (*p* = 0.52). **(G)** There was no difference between the groups with regards to Kansas City Cardiomyopathy Questionnaire Clinical Summary Score (*p* = 0.52). CI, confidence intercal; FRI, fluid restricted; IV, inverse variance; LFI, liberal fluid intatke; SD, standard deviation.

No significant differences were observed between fluid restriction and liberal fluid intake for all-cause mortality (RR: 1.71; 95% CI: 0.37–3.72; *P* = 0.79; I² = 1%; Figure 2A), HF rehospitalization (RR: 0.71; 95% CI: 0.46–1.10; *P* = 0.13; I² = 0%; Figure 2B), or thirst (WMD: 4.78; 95% CI: −6.72–16.28; *P* = 0.42; I² = 66%; Figure 2C). Fluid-restricted patients, however, had significantly lower daily fluid intake (WMD: −361.84 mL/day; 95% CI: −552.89 to −170.78; *P* < 0.001; I² = 0%; Figure 2D) and reduced adherence to the assigned regimen (WMD: 16.47%; 95% CI: 6.45–26.50; *P* = 0.001; I² = 0%; Figure 2E). Patient-reported outcomes showed no significant differences in the KCCQ overall summary score (KCCQ-OSS; WMD: 6.17; 95% CI: −12.54 to 24.87; *P* = 0.52; *I*^2^ = 75%; Figure 2F) as well as the KCCQ clinical summary score (KCCQ-CSS) (WMD: −6.7; 95% CI: −12.59 to −24.87; *P* = 0.52; *I*^2^ = 75%; Figure 2G) between the groups.

**Figure 3:**
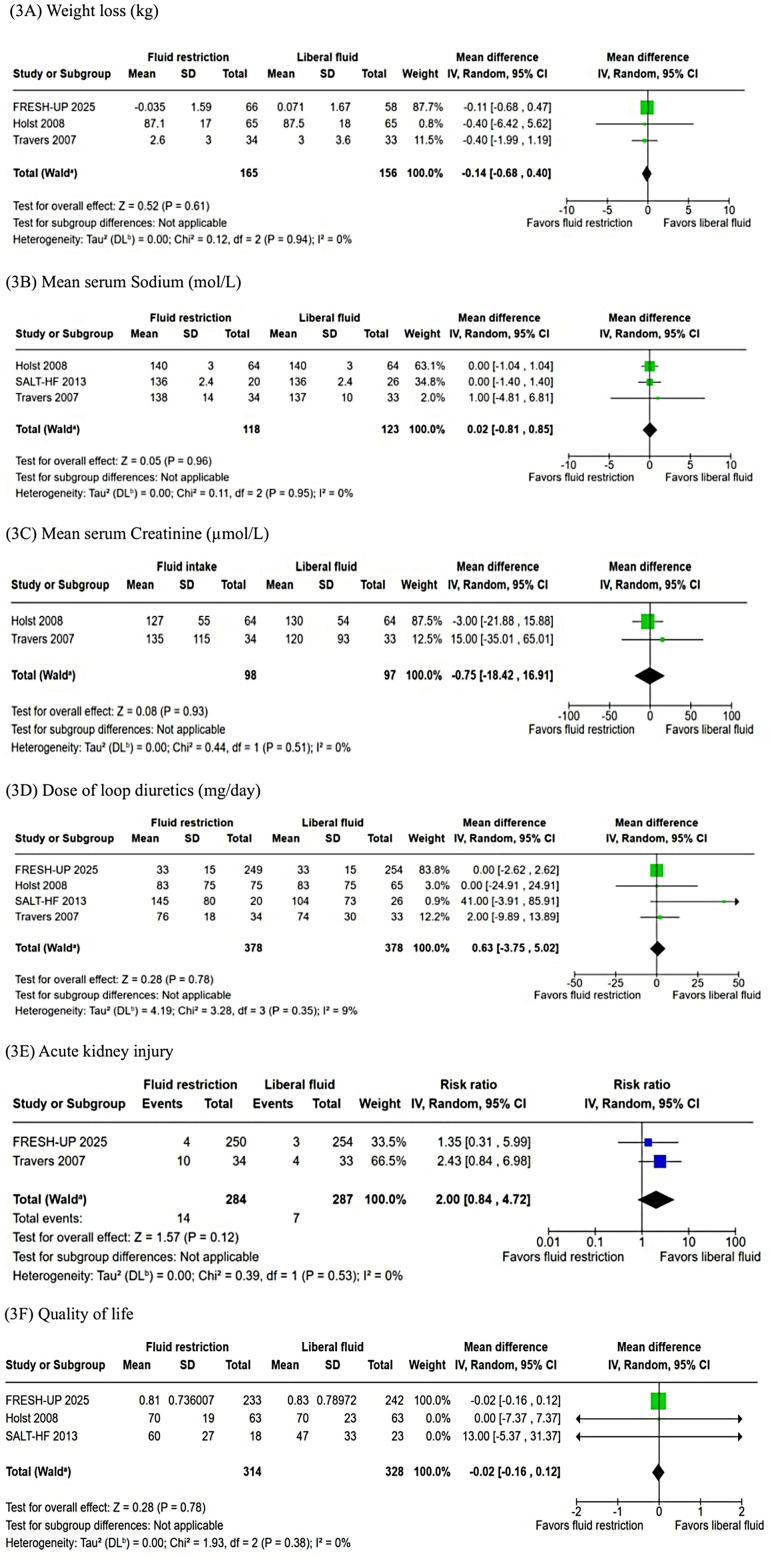
**(A)** The pooled analysis showed no significant difference in weight loss (kg) between FRI and LRI (*p* = 0.61). **(B)** There was no significant difference in mean serum sodium (mmol/L) between groups (*p* = 0.96). **(C)** There was no difference between FRI and LRI in mean serum creatinine (µmol/L) (*p* = 0.93). **(D)** The dose of loop diuretics (mg/day) was not significantly different between FRI and LRI (*p* = 0.78). **(E)** The incidence of acute kidney injury was not significantly different between the groups (*p* = 0.12). **(F)** There was no difference between FRI and LRI in QOL (*p* = 0.78). I, confidence intercal; FRI, fluid restricted; IV, inverse variance; LFI, liberal fluid intatke; SD, standard deviation.

Similarly, no significant differences were observed in weight loss (WMD: −0.14 kg; 95% CI: −0.68–0.40; *P* = 0.61; I² = 0%; Figure 3A), mean serum sodium levels (WMD: 0.02 mmol/L; 95% CI: −0.81–0.85; *P* = 0.96; I² = 0%; Figure 3B), mean serum creatinine (WMD: −0.75 µmol/L; 95% CI: −18.42–16.91; *P* = 0.93; I² = 0%; Figure 3C), or change in loop diuretic dose (WMD: 0.63 mg; 95% CI: −3.75–5.02; *P* = 0.78; I² = 9%; Figure 3D). The incidence of acute kidney injury was 4.9% in the fluid restriction group compared with 2.4% in the liberal fluid intake group, a difference that was not statistically significant (RR: 2.00; 95% CI: 0.84–4.72; *P* = 0.12; I² = 0%; Figure 3E). Similarly, QOL measures showed no significant differences between groups (WMD: −0.02; 95% CI: −0.16–0.12; *P* = 0.78; I² = 0%; Figure 3F).

### 3.3 Subgroup analyses

Fluid restriction was associated with significantly lower daily fluid intake in patients with reduced ejection fraction (EF < 40%) (WMD: −392.30 mL; 95% CI: −625.62 to −158.97; *P* = 0.001; I² = not applicable), but not in those with preserved EF (EF ≥ 40%) (WMD: −299.87 mL; 95% CI: −632.65–32.92; *P* = 0.08; I² = 0%; Supplementary Figure 1i). Similarly, fluid restriction led to significantly lower intake among patients with compensated HF (WMD: −358.64 mL; 95% CI: −552.35 to −164.93; *P* < 0.001; I² = 0%), but not in those with decompensated HF (WMD: −476.00 mL; 95% CI: −1,633.71–681.71; *P* = 0.42; I² = not applicable; Supplementary Figure 1ii). In addition, patients with baseline fluid intake ≥1.5 L/day demonstrated a significant reduction with fluid restriction (WMD: −552.35 mL; 95% CI: −925.55 to −179.15; *P* < 0.001; I² = 0%), whereas no significant difference was observed among those with baseline intake <1.5 L/day (WMD: −476.35 mL; 95% CI: −1,633.71–681.71; *P* = 0.42; I² = not applicable; Supplementary Figure 1iii).

There was no significant difference in thirst levels between the groups in patients with EF < 40% (WMD: 6.29; 95% CI: −17.95–30.54; *P* = 0.61; I² = 71%) or those with EF ≥ 40% (WMD: 1.70; 95% CI: 0.19–3.21; *P* = 0.03; I² = not applicable; Supplementary Figure 2i). Similarly, thirst did not differ significantly according to HF status, whether decompensated (WMD: −8.00; 95% CI: −30.76–14.76; *P* = 0.49; I² = not applicable) or compensated (WMD: 7.89; 95% CI: −6.83–22.61; *P* = 0.29; I² = 80%; Supplementary Figure 2ii). In addition, no significant difference was observed in thirst between patients with a baseline fluid intake ≥1.5 L/day (WMD: 7.89; 95% CI: −6.83–22.61; *P* = 0.29; I² = 80%) and those with <1.5 L/day (WMD: −8.00; 95% CI: −30.76–14.76; *P* = 0.49; I² = not applicable; Supplementary Figure 2iii).

### 3.4 Quality assessment

**Figure 4:**
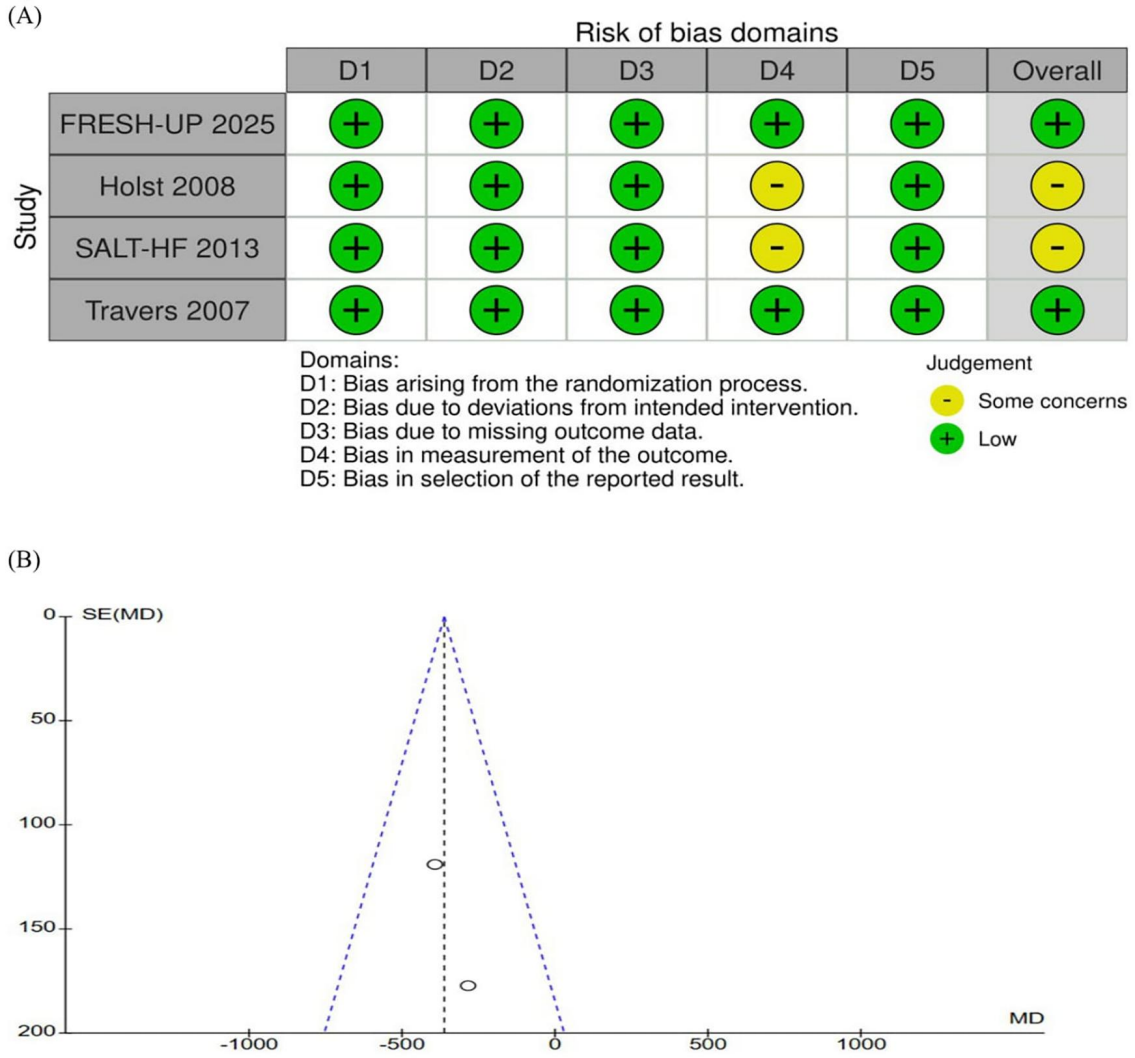
**(A)** Critical appraisal of randomized controlled trials according to the cochrane collaboration tool for assessing risk of bias in randomized trials (RoB2). None of the studies were considered at high risk of bias through the RoB2 tool **(B)** funnel plot analysis of the daily total fluid intake (mL/day) shows no evidence of publication bias. SE, standard erro; MD, mean difference.

The quality appraisal of the included RCTs is presented in Figure 4A. Overall, the studies were judged to have a low risk of bias across most domains, except for some concerns in the measurement of outcomes (5, 6). Notably, the trial by Holst et al., which employed a crossover design, appropriately evaluated the potential carryover effects (5). Although fewer than 10 studies were included, which may limit the statistical power of publication bias assessments, visual inspection of the funnel plot revealed a symmetrical distribution of study weights around the pooled effect estimate, suggesting no publication bias (Figure 4B).

### 3.5 Sensitivity analysis

We performed leave-one-out analyses for all outcomes. Overall, excluding individual studies did not change the statistical significance of any of the outcomes. However, for thirst, the exclusion of the SALT-HF trial reduced heterogeneity from I² = 66%–I² = 0% and shifted the pooled effect to significantly favor liberal fluid intake (*P* = 0.03) (Supplementary Table 2) (6). This effect was likely driven by the functional status of patients in this trial, who represented the sickest subgroup. The exclusion of other studies had no notable influence on heterogeneity or effect size.

## 4 Discussion

This meta-analysis of 4 RCTs, including 747 patients with HF, compared fluid restriction with liberal fluid intake. The main findings were as follows: (1) no significant difference in all-cause mortality or HF rehospitalization; (2) fluid restriction lead to reduced total fluid intake (3) liberal fluid intake was associated with higher adherence; and (4) no significant differences were observed in KCCQ-OSS, KCCQ-CSS, QoL, weight change, or loop diuretic requirements.

Fluid management remains one of the most debated aspects of HF care. While fluid restriction has traditionally been recommended to mitigate congestion, the supporting evidence has been inconsistent and often inconclusive. Although restriction predictably reduces daily fluid intake, it has not consistently translated into improved clinical outcomes (1–3, 8). Conversely, liberal fluid intake has been hypothesized to improve patient comfort, adherence, and hydration without compromising safety (5). Notably, recent high-quality evidence, including the FRESH-UP trial, has demonstrated no increased risk associated with a more liberal approach to fluid intake (11).

In this context, our meta-analysis provides an updated synthesis of randomized data, reinforcing that routine fluid restriction does not improve mortality, rehospitalization, QOL, or functional capacity. This reinforces the lack of clinical benefit from routine fluid restriction in stable HF patients, despite achieving a modest reduction in fluid intake. These findings are consistent with a previous meta-analysis by Li et al., which similarly reported no differences in rehospitalization or mortality between fluid restriction and liberal fluid intake (9). Furthermore, another meta-analysis incorporating trial sequential analysis confirmed the absence of benefit in reducing clinical events with fluid restriction (10). The lack of significant difference in the thirst intensity (11), QoL (5, 6, 11) with fluid restriction was already known from previous RCTs. This may reflect the multifactorial nature of HF, heterogeneity in baseline fluid status, and the influence of other clinical and psychosocial factors that may contribute to patients’ overall well-being.

Although fluid restriction resulted in a modest but statistically significant reduction in fluid intake (WMD: 362 mL/day) among patients with HF, this did not translate into meaningful clinical or symptomatic benefits in our study. Concerns however, remained that excessive fluid restriction may impair renal perfusion and activate neurohormonal pathways, potentially leading to worsened clinical outcomes (4, 15). Our meta-analysis did not find a significant difference in mean serum creatinine levels between fluid restriction and liberal intake groups. This contrasts with findings from recent meta-analyses that suggested otherwise (9, 10). For instance, the meta-analysis by Hsu et al. reported a significant increase in mean serum creatinine with fluid restriction. A likely explanation for this discrepancy lies in the substantial heterogeneity of their findings and their inclusion of observational studies, whereas our analysis was restricted to RCTs, which may provide more robust and less biased estimates. Conversely, patients randomized to liberal fluid intake demonstrated better adherence to the prescribed regimen, highlighting the potential practical advantage of less restrictive strategies in real-world HF care. Adherence is a critical factor in chronic disease management, as poor adherence can undermine the effectiveness of therapy and patient outcomes.

Our findings indicate that while fluid restriction reduces intake in the fluid restricted group, it does not improve KCCQ-CSS, KCCQ-OSS, hospitalization, mortality, renal function, thirst, or QoL. The KCCQ, a validated measure of HF symptoms and QoL, underscores the patient-centered impact of the interventions. These results support individualized fluid management based on patient status rather than routine restriction and may inform future guideline updates to emphasize personalized strategies that improve adherence, comfort, and clinical outcomes. Additionally, the complex interplay between HF and renal function underscores the need for careful fluid management. The ongoing FLUID-HF trial, a 12-week non-inferiority study incorporating measures such as lung ultrasound B-lines, is expected to provide more definitive evidence on optimal fluid management strategies in HF underscoring the need for robust data to guide patient-centered care “(ClinicalTrials.gov ID: NCT05931614).”

## 5 Strengths and limitations

This meta-analysis has several strengths, including the inclusion of a recent RCT, comprehensive sensitivity and subgroup analyses, focus on both clinical and patient-centered outcomes, and the exclusive evaluation of fluid restriction strategies in HF.

However, some limitations of this study should be acknowledged. First, the number of included RCTs was small, and most studies had limited sample sizes with fewer reported outcomes. This may have reduced the statistical power to detect clinically significant differences between the groups. Although we applied a random-effects model and performed sensitivity analyses to assess the robustness of our findings, the potential for imprecision remains, and the results should be interpreted cautiously pending further high-quality, adequately powered trials. Second, moderate to high heterogeneity was observed for several outcomes, including the KCCQ-OSS, KCCQ-CSS, and thirst. In addition, the duration of our search and the substantial time gap between the most recent trial (FRESH-UP) and earlier studies may reflect differences in background therapy and standard of care, potentially contributing to clinical heterogeneity. Notably, the leave-one-out sensitivity analyses revealed that excluding the SALT-HF study resulted in a statistically significant and consistent reduction in thirst, favoring liberal fluid intake. This finding suggests that the overall estimate was highly sensitive to this study, underscoring the need for cautious interpretation. Third, owing to the nature of the intervention, blinding of participants and healthcare providers was not feasible in most trials, introducing a potential risk of performance and detection bias, particularly for subjective outcomes such as thirst and QoL. Fourth, several studies did not report key biochemical endpoints, such as natriuretic peptide levels, thereby limiting our ability to assess potential mechanistic effects and precluding more detailed subgroup analyses. Finally, it should be noted that all the included trials were conducted in Europe and the USA, which may limit the generalizability of the findings to other populations due to potential racial and ethnic differences in salt and water handling.

## 6 Conclusion

In this meta-analysis of RCTs evaluating optimal fluid management in patients with HF, fluid restriction was associated with lower daily fluid intake compared with liberal fluid intake. In contrast, adherence was significantly higher in the liberal fluid intake group. No significant differences were observed between groups in KCCQ-CSS, KCCQ-OSS, hospitalization, mortality, worsening renal function, thirst, or overall quality of life. These findings may inform clinical decision-making and support a more individualized approach to fluid management in patients with HF.

## Data Availability

The original contributions presented in the study are included in the article/Supplementary Material, further inquiries can be directed to the corresponding author.

## References

[1] McDonagh TA Metra M Adamo M Gardner RS Baumbach A Böhm M 2021 ESC guidelines for the diagnosis and treatment of acute and chronic heart failure. Eur Heart J. (2021) 42:4901. 10.1093/eurheartj/ehab368 34922348

[2] Tsao CW Aday AW Almarzooq ZI Alonso A Beaton AZ Bittencourt MS Heart disease and stroke statistics-2022 update: a report from the American Heart Association. Circulation. (2022) 145(8):e153––e639. 10.1161/CIR.0000000000001052 35078371

[3] Cosentino N Marenzi G Muratori M Magrì D Cattadori G Agostoni P. Fluid balance in heart failure. Eur J Prev Cardiol. (2023) 30:ii9–15. 10.1093/eurjpc/zwad166 37819223

[4] Mullens W Damman K Dhont S Banerjee D Bayes-Genis A Cannata A Dietary sodium and fluid intake in heart failure. A clinical consensus statement of the heart failure association of the ESC. Eur J Heart Fail. (2024) 26(4):730–41. 10.1002/ejhf.3244 38606657

[5] Holst M Strömberg A Lindholm M Willenheimer R. Liberal versus restricted fluid prescription in stabilised patients with chronic heart failure: result of a randomised cross-over study of the effects on health-related quality of life, physical capacity, thirst and morbidity. Scand Cardiovasc J. (2008) 42:316–22. 10.1080/14017430802071200 18609051

[6] Albert NM Nutter B Forney J Slifcak E Tang WHW. A randomized controlled pilot study of outcomes of strict allowance of fluid therapy in hyponatremic heart failure (SALT-HF). J Card Fail. (2013) 19:1–9. 10.1016/j.cardfail.2012.11.007 23273588

[7] Travers B O’Loughlin C Murphy NF Ryder M Conlon C Ledwidge M Fluid restriction in the management of decompensated heart failure: no impact on time to clinical stability. J Card Fail. (2007) 13:128–32. 10.1016j.cardfail.2006.10.012 17395053 10.1016/j.cardfail.2006.10.012

[8] Heidenreich PA Bozkurt B Aguilar D Allen LA Byun JJ Colvin MM 2022 AHA/ACC/HFSA guideline for the management of heart failure: a report of the American College of Cardiology/American Heart Association joint committee on clinical practice guidelines. J Am Coll Cardiol. (2022) 79(17):e263–421. 10.1016/j.jacc.2021.12.012 35379503

[9] Li Y Fu B Qian X. Liberal versus restricted fluid administration in heart failure patients. A systematic review and meta-analysis of randomized trials. Int Heart J. (2015) 56:192–5. 10.1536/ihj.14-288 25740394

[10] Hsu SM Lin YH Lin YC Liu SJ Liu CJ Hung CL Fluid intake impact on heart failure: systematic review and meta-analysis with trial sequential analysis. J Formos Med Assoc. (2024) 124(7):650–9. 10.1016/j.jfma.2024.11.017 39603913

[11] Herrmann JJ Brunner-La Rocca HP Baltussen LEHJM Beckers-Wesche F Bekkers SCAM Bellersen L Liberal fluid intake versus fluid restriction in chronic heart failure: a randomized clinical trial. Nat Med. (2025) 31:2062–8. 10.1038/s41591-025-03628-4 40159556

[12] Page MJ McKenzie JE Bossuyt PM Boutron I Hoffmann TC Mulrow CD The PRISMA 2020 statement: an updated guideline for reporting systematic reviews. Br Med J. (2021):372. 10.1136/BMJ.N71 PMC8005924 33782057

[13] Sterne JAC Savović J Page MJ Elbers RG Blencowe NS Boutron I Rob 2: a revised tool for assessing risk of bias in randomised trials. Br Med J. (2019) 366:I4898. 10.1136/BMJ.L4898 31462531

[14] Higgins JPT Thomas J Chandler J Cumpston M Li T Page MJ Cochrane handbook for systematic reviews of interventions version 6.3 (updated February 2022). Cochrane. (2022). Available online: www.training.cochrane.org/handbook

[15] Colin-Ramirez E Arcand J Saldarriaga C Ezekowitz JA. The current state of evidence for sodium and fluid restriction in heart failure. Prog Cardiovasc Dis. (2024) 82:43–54. 10.1016/j.pcad.2024.01.004 38215917

