# Correction: Optimal fluid management strategies in patients with heart failure: a systematic review and meta-analysis of randomized controlled trials

**DOI:** 10.3389/fcvm.2026.1791038

**Published:** 2026-03-30

**Authors:** Umar G. Adamu, Blessing Muponda, Nqoba Tsabedze

**Affiliations:** Division of Cardiology, Department of Internal Medicine, School of Clinical Medicine, Faculty of Health Sciences, University of the Witwatersrand, Johannesburg, South Africa

**Keywords:** heart failure, fluid restriction, liberal fluid intake, hospitalization, mortality, quality of life

There was a mistake in Figures 2F and 2G as published. In the originally published article, Figure 2G was incorrectly labeled as Figure 2F, and Figure 2G was mistakenly replaced with a duplicate of Figure 2D. The corrected versions of Figures 2F and 2G appear below.

**Figure 2F:**
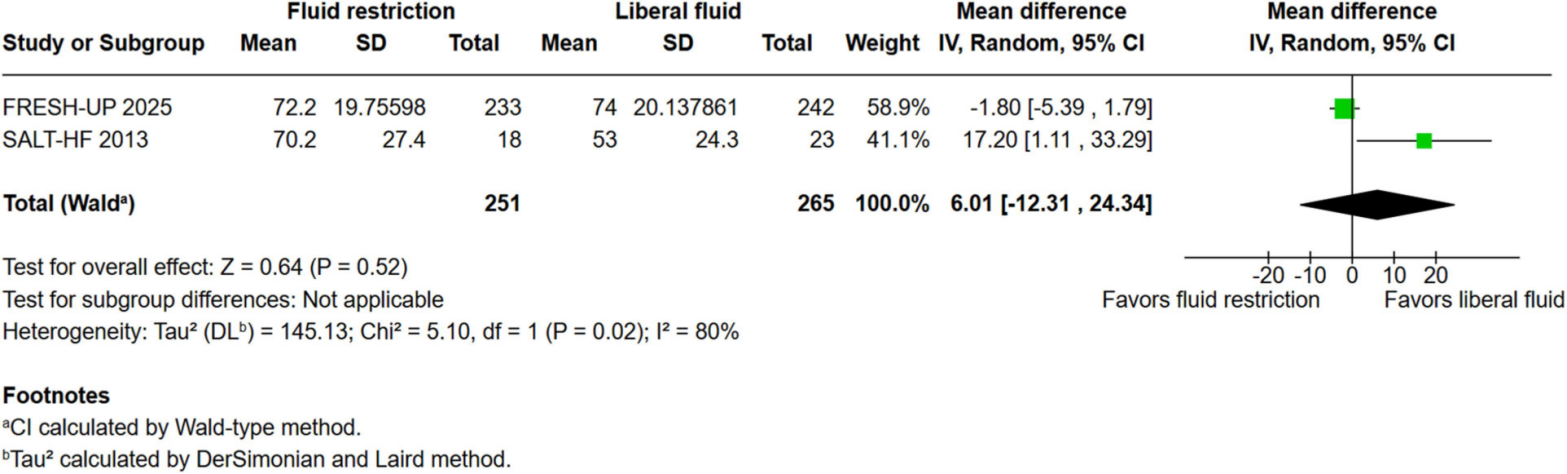
Kansas City cardiomyopathy questionnaire overall summary score.

**Figure 2G:**
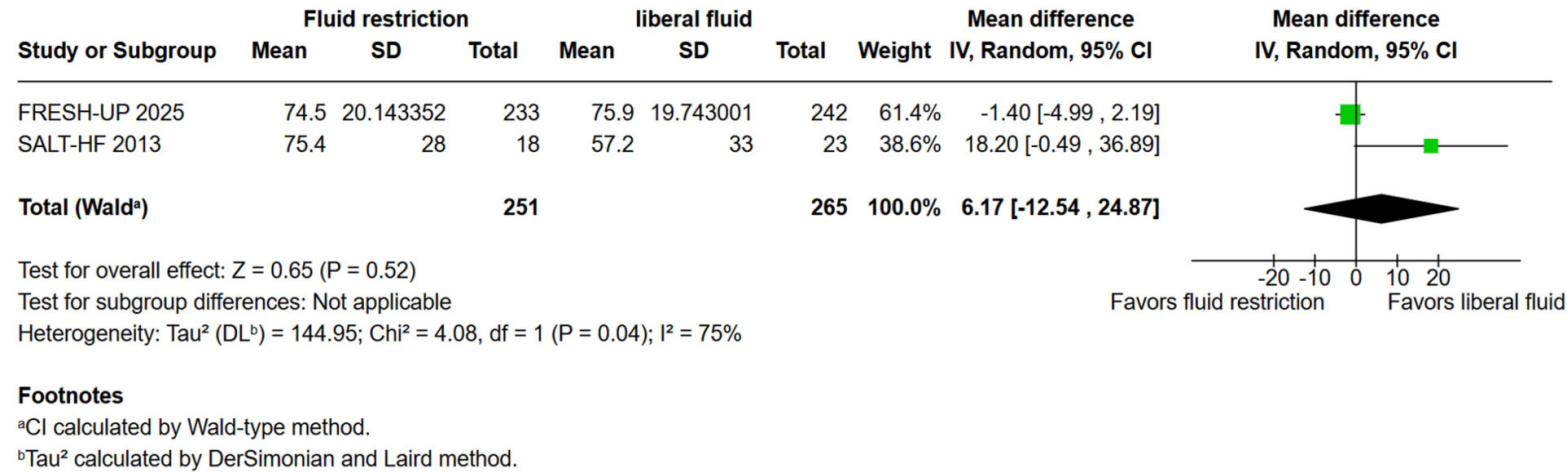
Kansas City cardiomyopathy questionnaire clinical summary score.

There was a mistake in the caption of Figure 2G. The published caption is:

“**(G)** The Kansas City Cardiomyopathy Questionnaire Clinical Summary Score was significantly lower in the FRI group (*p* < 0.001).”

The corrected caption of Figure 2G is:

“**(G)** There was no difference between the groups with regards to Kansas City Cardiomyopathy Questionnaire Clinical Summary Score (*p* = 0.52).”

The following sentence from the results section in the **Abstract** was erroneously worded as:

“Patients in the fluid restriction group had significantly lower fluid intake (WMD: −361.84 mL/day; 95% CI: −552.89 to −170.78; *p* < 0.001), lower Kansas City Cardiomyopathy Questionnaire (KCCQ) Clinical summary score (WMD: −361.84; 95% CI: −552.89 to −170.78; *P* < 0.001), and lower adherence (WMD: 16.47; 95% CI: 6.45–26.50; *p* = 0.001). No significant differences were observed between groups in terms of acute kidney injury, weight loss, or patient-reported quality of life.”

The corrected sentence is:

“Patients in the fluid restriction group had significantly lower fluid intake (WMD: −361.84 mL/day; 95% CI: −552.89 to −170.78; *p* < 0.001) and lower adherence (WMD: 16.47; 95% CI: 6.45–26.50; *p* = 0.001). No significant differences were observed between groups in terms of acute kidney injury, weight loss, or patient-reported quality of life, and Kansas City Cardiomyopathy Questionnaire (KCCQ) Clinical summary score.”

The following sentence from the conclusion section in the **Abstract** was erroneously worded as:

“Conclusions: In this meta-analysis, fluid restriction significantly reduced total fluid intake but did not improve clinical outcomes in patients with HF. Adherence and KCCQ clinical summary scores were higher with liberal fluid intake. These findings support an individualized approach to fluid management in patients with HF.”

The corrected sentence is:

“Conclusions: In this meta-analysis, fluid restriction significantly reduced total fluid intake but did not improve clinical outcomes in patients with HF. Adherence was higher with liberal fluid intake. These findings support an individualized approach to fluid management in patients with HF.”

A correction has been made to **3 Results,** *3.2 Outcomes*, paragraph 1. The sentence previously read:

“Patient-reported outcomes showed no significant differences in the KCCQ overall summary score (KCCQ-OSS; WMD: 6.17; 95% CI: −12.54–24.87; *P* = 0.52; I² = 75%; Figure 2F). However, the KCCQ clinical summary score (KCCQ-CSS) was significantly different in the fluid restricted group (WMD: −361.84; 95% CI: −552.89 to −170.78; *P* < 0.001; I² = 0%; Figure 2G).”

The corrected sentence is:

“Patient-reported outcomes showed no significant differences in the KCCQ overall summary score (KCCQ-OSS; WMD: 6.17; 95% CI: −12.54 to 24.87; *P* = 0.52; *I*^2^ = 75%; Figure 2F) as well as the KCCQ clinical summary score (KCCQ-CSS) (WMD: −6.7; 95% CI: −12.59 to −24.87; *P* =0.52; *I*^2^ = 75%; Figure 2G) between the groups.”

A correction has been made to **4 Discussion**, paragraph 1. The sentence previously read:

“This meta-analysis of 4 RCTs, including 747 patients with HF, compared fluid restriction with liberal fluid intake. The main findings were as follows: (1) no significant difference in all-cause mortality or HF rehospitalization; (2) fluid restriction reduced total fluid intake and KCCQ-CSS; (3) liberal fluid intake was associated with higher adherence; and (4) no significant differences were observed in KCCQ-OSS, QoL, weight change, or loop diuretic requirements.”

The corrected sentence is:

“This meta-analysis of 4 RCTs, including 747 patients with HF, compared fluid restriction with liberal fluid intake. The main findings were as follows: (1) no significant difference in all-cause mortality or HF rehospitalization; (2) fluid restriction lead to reduced total fluid intake (3) liberal fluid intake was associated with higher adherence; and (4) no significant differences were observed in KCCQ-OSS, KCCQ-CSS, QoL, weight change, or loop diuretic requirements.”

A correction has been made to **4 Discussion**, paragraph 5. The sentence ¨previously read:

“Our findings indicate that while fluid restriction reduces intake and KCCQ-CSS, it does not improve hospitalization, mortality, renal function, thirst, or QoL”.

The corrected sentence is:

“Our findings indicate that while fluid restriction reduces intake in the fluid restricted group, it does not improve KCCQ-CSS, KCCQ-OSS, hospitalization, mortality, renal function, thirst, or QoL.”

A correction has been made to 6 **Conclusion**. The sentence previously read:

“In contrast, adherence and KCCQ clinical summary scores were significantly higher in the liberal fluid intake group. No significant differences were observed between groups in hospitalization, mortality, worsening renal function, thirst, or overall quality of life.”

The corrected sentence is:

“In contrast, adherence was significantly higher in the liberal fluid intake group. No significant differences were observed between groups in KCCQ-CSS, KCCQ-OSS, hospitalization, mortality, worsening renal function, thirst, or overall quality of life.”

The original version of this article has been updated.

