# Supplementary material for: Optimal fluid management strategies in patients with heart failure: a systematic review and meta-analysis of randomized controlled trials
Source: Front Cardiovasc Med. 2025 Oct 31;12:1636862. doi: 10.3389/fcvm.2025.1636862 (PMC12615477; doi:10.3389/fcvm.2025.1636862)
Supplement: Supplementary file 1 [file Table1.docx]

Supplementary Material

**Umar G. Adamu, Blessing Muponda, Nqoba Tsabedze**

**Optimal fluid management strategies in patients with heart failure: A systematic review and meta-analysis of randomized controlled trials**

Supplementary Table 1. Detailed search **strategy** for each of the databases

Supplementary Table 2. Leave-one-out sensitivity analysis of the studies that reported on thirst.

Supplementary Figure 1. Subgroup analysis of total fluid intake.

Supplementary Figure 2. Subgroup analysis of thirst.

**Supplementary Table 1:** Detailed search for each of the databases

| **PubMed** | ("Heart failure" OR "Heart Failure"[Mesh] OR "Chronic heart failure" OR "congestive heart failure" OR "compensated heart failure" OR HFrEF OR HFpEF OR HFmrEF) AND ("Fluid therapy" OR "fluid therapy"[Mesh] OR "fluid intake" OR "water intake" OR hydration OR "fluid management" OR drinking OR "nutritional support"[Mesh] OR "nutrition therapy" OR "diet therapy" OR "diet intervention" OR "nutrition intervention" OR "dietary intervention" OR fluid ) AND ("Fluid restriction" OR "restricted fluid" OR "liberal fluid" OR "increased fluid intake" OR "water deprivation") |
| --- | --- |
| **Embase** | ("Heart failure" OR "Chronic heart failure" OR "congestive heart failure" OR "compensated heart failure" OR HFrEF OR HFpEF OR HFmrEF) AND ("Fluid therapy" OR "fluid intake" OR "water intake" OR hydration OR "fluid management" OR drinking OR "nutritional support" OR "nutrition therapy" OR "diet therapy" OR "diet intervention" OR "nutrition intervention" OR "dietary intervention" OR fluid ) AND ("Fluid restriction" OR "restricted fluid" OR "liberal fluid" OR "increased fluid intake" OR "water deprivation") |
| **Cochrane** | ("Heart failure" OR "Chronic heart failure" OR "congestive heart failure" OR "compensated heart failure" OR HFrEF OR HFpEF OR HFmrEF) AND ("Fluid therapy" OR "fluid intake" OR "water intake" OR hydration OR "fluid management" OR drinking OR "nutritional support" OR "nutrition therapy" OR "diet therapy" OR "diet intervention" OR "nutrition intervention" OR "dietary intervention" OR fluid ) AND ("Fluid restriction" OR "restricted fluid" OR "liberal fluid" OR "increased fluid intake" OR "water deprivation") AND (randomized OR random OR RCT) |

**Supplementary Table 2:** Leave-one-out sensitivity analysis for thirst

|  | Mean difference | 95% CI | P value | I^2^ (%) |
| --- | --- | --- | --- | --- |
| Omitting FRESH-UP | 6.29 | -17.95-30.54 | 0.61 | 77 |
| Omitting Holst | 1.66 | 0.15-3.17 | 0.03 | 0 |
| Omitting SALT-HF | 7.89 | -16.03-22.81 | 0.29 | 80 |

CI, confidence interval

**(1) Total fluid intake (mL/day**)
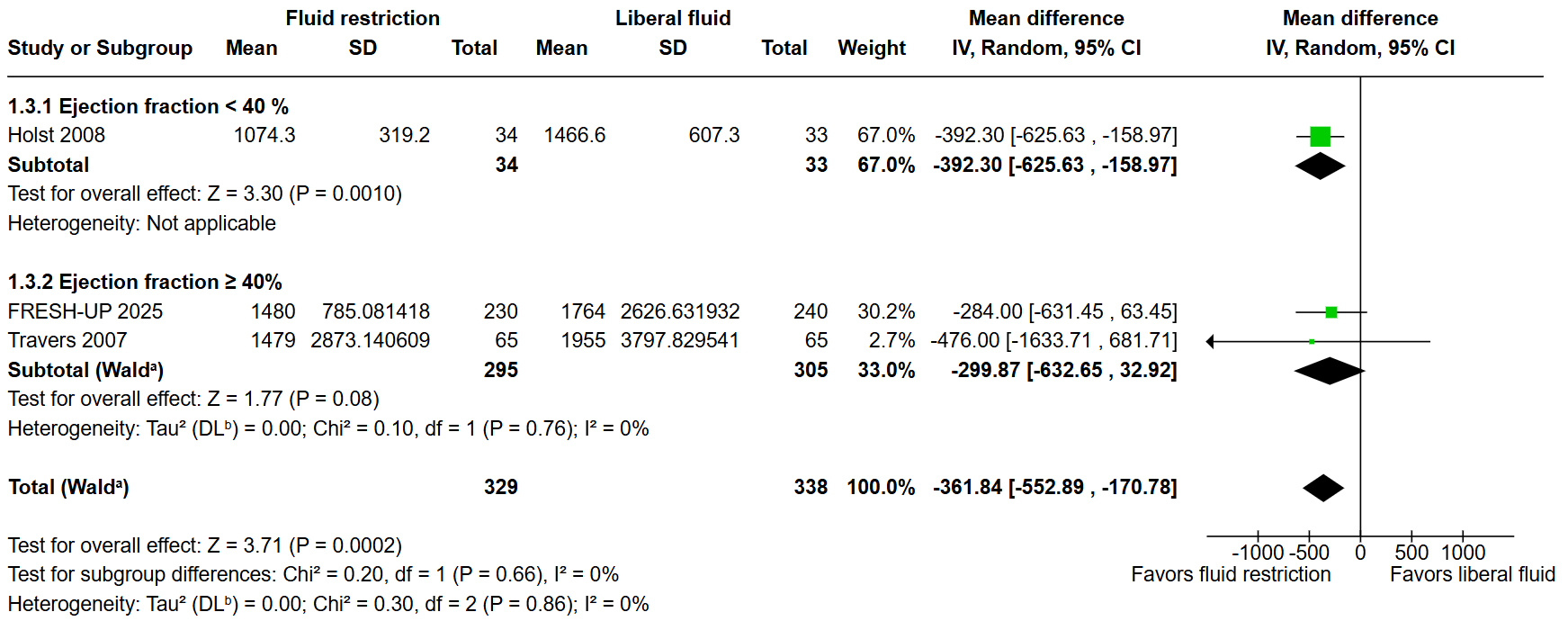


**(i) Ejection fraction (%)**


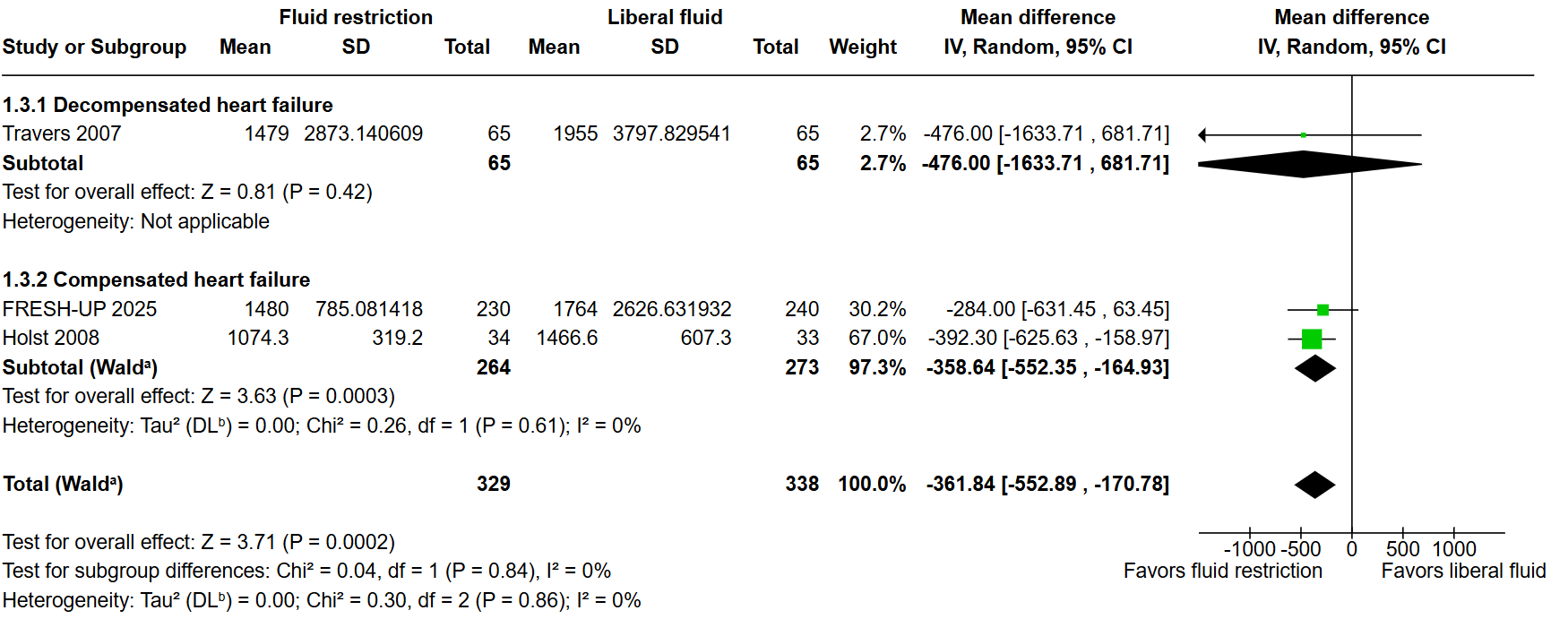


1. **Heart failure status**


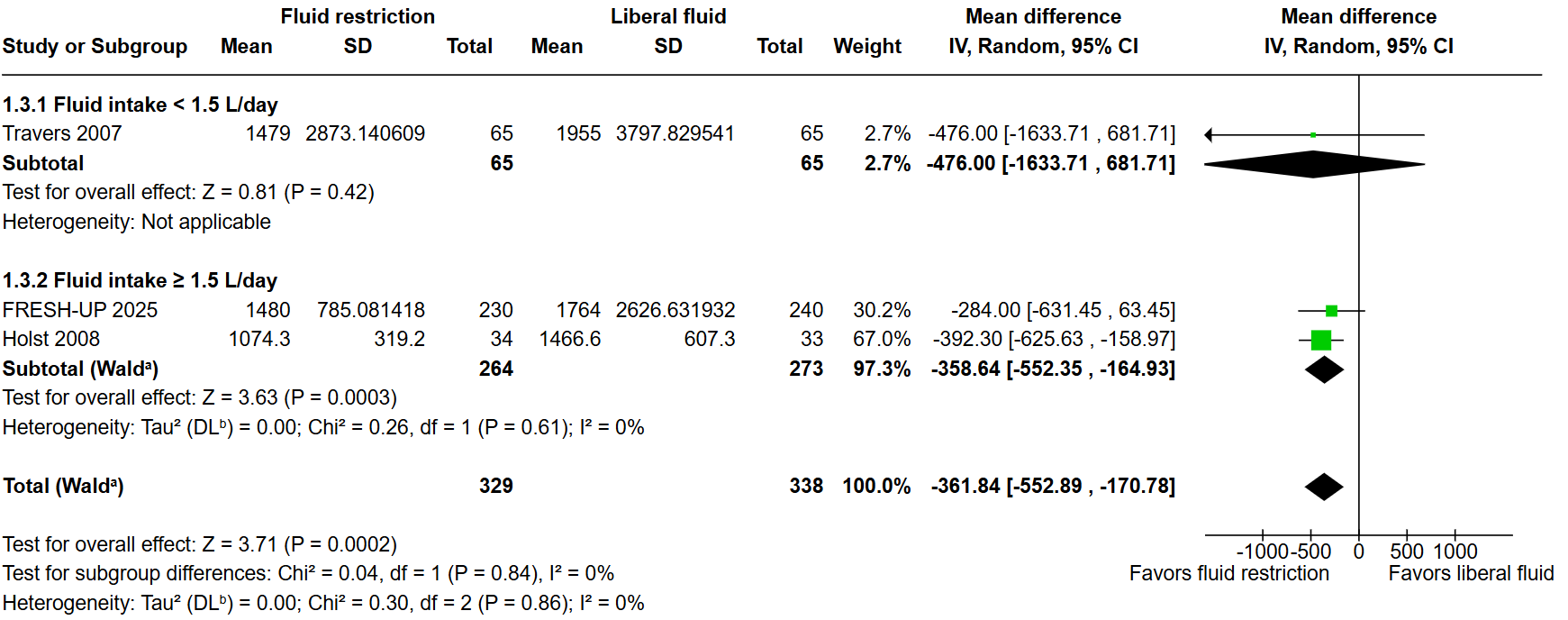


(iii) Fluid allowed per day (mL/day)

**(2) Thirst**


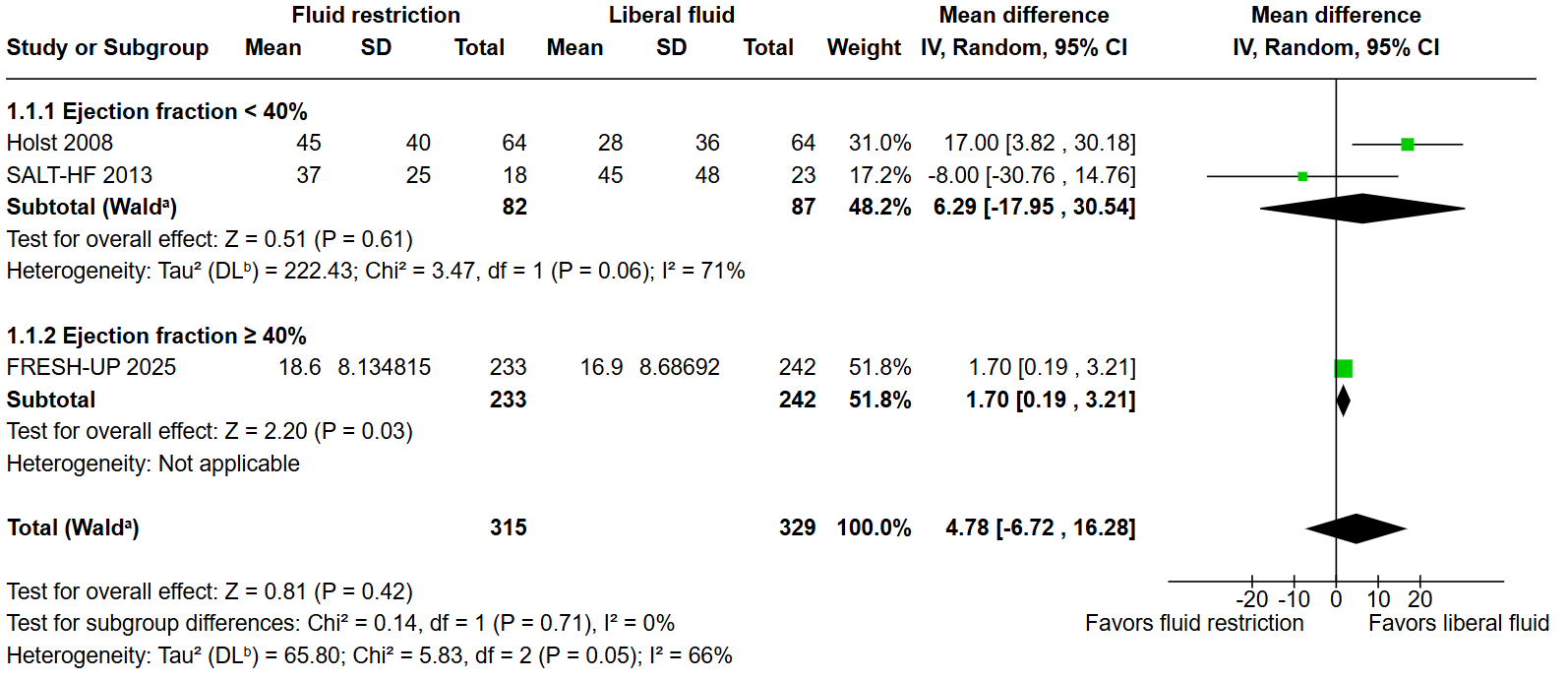


**(i) Ejection fraction (%)**


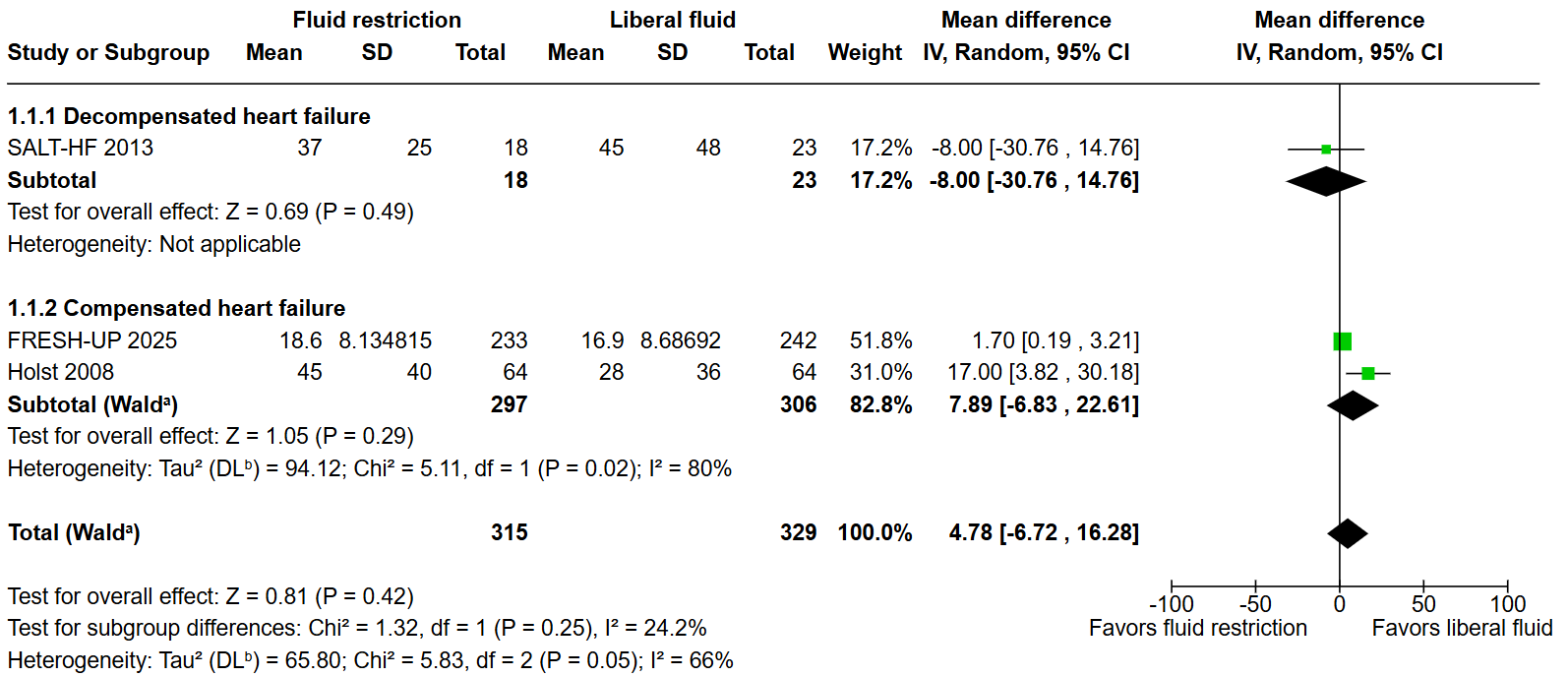


(ii) **Heart failure status**


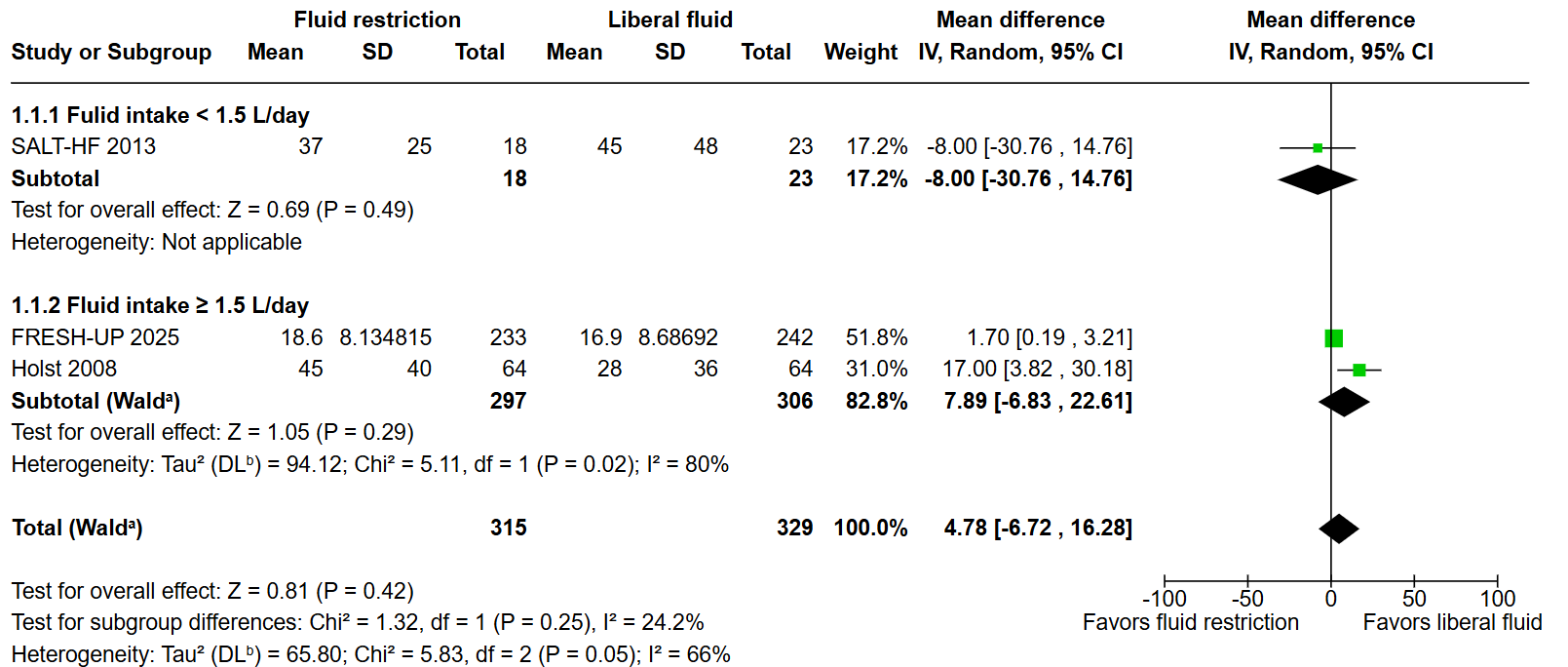


(iii) Fluid allowed per day (mL/day)

**Supplementary Figure 1 Total fluid intake (i) ejection fraction (%); (ii) Heart failure status; and (iii) Fluid allowed per day (mL/day)**

**Supplementary Figure 2 Thirst (i) ejection fraction (%); (ii) Heart failure status; and (iii) Fluid allowed per day (mL/day). CI. Confidence interval; IV. Inverse variance; SD. Standard deviation.**
